# Fermented Unpolished Black Rice (*Oryza sativa* L.) Inhibits Melanogenesis via ERK, p38, and AKT Phosphorylation in B16F10 Melanoma Cells

**DOI:** 10.4014/jmb.2003.03019

**Published:** 2020-05-08

**Authors:** Orrarat Sangkaew, Chulee Yompakdee

**Affiliations:** Department of Microbiology, Faculty of Science, Chulalongkorn University, Bangkok 10330, Thailand

**Keywords:** Fermented rice, unpolished black rice, Hom Nin rice, *Oryza sativa* L., melanogenesis inhibition, melanogenesis-related proteins

## Abstract

Melanin is a major factor that darkens skin color as one of the defense systems to prevent the harmful effects of UV light. However, darkened skin from the localized or systemic accumulation of melanin is viewed in many cultures as an esthetic problem. Consequentially, searching for anti- melanogenic agents from natural sources is very popular worldwide. Previous screening of fermented rice products, obtained from various rice cultivars fermented with different sources of loog-pang (Thai traditional fermentation starter), revealed that the highest ability to reduce the melanin content in B16F10 melanoma cells was from unpolished black rice fermented with a defined starter mixture of microbes isolated from loog-pang E11. The aim of this study was to investigate the mechanism of the fermented unpolished black rice (FUBR) on the inhibition of melanogenesis in B16F10 melanoma cells. The strongest reduction of cellular melanin content was found in the FUBR sap (FUBRS). The melanin reduction activity was consistent with the significant decrease in the intracellular tyrosinase activity. The FUBRS showed no cytotoxic effect to B16F10 melanoma or Hs68 human fibroblast cell lines. It also significantly reduced the transcript and protein expression levels of tyrosinase, tyrosinase-related protein 1 (TYRP-1), TYRP-2, and microphthalmia-associated transcription factor. Furthermore, it induced a significantly increased level of phosphorylated ERK, p38 and Akt signaling pathways, which likely contributed to the negative regulation of melanogenesis. From these results, a model for the mechanism of FUBRS on melanogenesis inhibition was proposed. Moreover, these results strongly suggested that FUBRS possesses anti- melanogenesis activity with high potential for cosmeceutical application as a skin depigmenting agent.

## Introduction

Melanin is a skin pigment that plays an important role in protection from the harmful effects caused by ultra- violet (UV) light. Overproduction and accumulation of melanin can result in pigmented patches and skin discolorations, such as chloasma, solar lentigo and freckles, that lead to perceived esthetic problems [1, 2].

The production of melanin pigment, also called “melanogenesis,” is a complicated process regulated by various melanogenesis enzymes, such as tyrosinase, and tyrosinase-related protein 1 (TYRP1) and TYRP2. Among these enzymes, tyrosinase is the essential enzyme in melanogenesis. It catalyzes two initial steps in this process; namely hydroxylation of L-tyrosine into 3,4-dihydroxyphenylalanine (L-DOPA) and the oxidation of L-DOPA into dopaquinone. These melanogenesis enzymes are regulated by microphthalmia-associated transcription factor (MITF) [3-5].

In addition, melanogenesis is modulated by mitogen-activated protein kinase (MAPK) signaling via extracellular signal-regulated kinases (ERK) and p38 MAPK. The phosphorylation of ERK (Thr202/Tyr204) and p38 (Thr180/Tyr182) leads to MITF degradation, which plays a pivotal role in suppressing melanin production [6-8]. The phosphatidylinositol 3-kinase (PI3K)/Akt signaling pathway is also known to be involved in the regulation of melanogenesis, where the phosphorylation of Akt leads to negative regulation of melanogenesis [9, 10]. Additionally, UV light radiation causes the synthesis of reactive oxygen species (ROS) that are also involved in the regulation of melanin synthesis. Hence, ROS scavengers and inhibitors of ROS generation may down-regulate melanogenesis [11].

Currently, known depigmenting agents, such as kojic acid, arbutin and linoleic acid, are used as cosmetic agents for skin whitening. However, these compounds should be used at a limited dosage due to their cytotoxicity and carcinogenic potential [12]. Accordingly, safer and more effective whitening agents are needed in the cosmetic market.

Natural materials are potentially suitable for the development of effective and safer skin whitening agents in skin care products. Rice (*Oryza sativa* L.) is one such interesting natural material and is the most important cereal food crop in many Asian countries, including in Thailand [13]. It has been proposed as a potential source of bioactive compounds for health benefits, where a variety of rice cultivars are reported to have medicinal effects [14], such as protection against cytotoxicity, anti-neurodegenerative activity [15], glycogen phosphorylase inhibition [16], and antioxidative activity [17]. Recently, various cultivars of rice have been shown to be able to stimulate melanogenesis [18]. Fermentation is a biotechnological process used as an alternative way to modify, including improve, the biological functionalities of compounds in substrates. Many reports have demonstrated that fermentation can not only enhance the biological activity of a substrate, but can also alter the original bioactive compounds, resulting in a new biological activity [19-21].

Khao-mak a healthy dessert from Thailand, is produced from rice fermented with a microbial starter culture called “loog-pang.” It is considered to promote growth in malnourished children, activate bacterial activity, and is used as a dietary supplement. Hence, fermented rice is an interesting natural resource. *loog-pang* contains yeasts, molds and lactic acid bacteria (LAB), and is mixed with rice to produce fermented rice products [22]. In our preliminary study, various fermented rice samples prepared from different types of rice cultivar and fermented with different sources of loog-pang for 12 d were screened for their ability to reduce the melanin content in B16F10 melanoma cells. The product from unpolished black rice (UBR; Hom Nin rice) fermented with loog-pang E11 showed the highest reduction activity of melanin content in B16F10 melanoma cells. Thus, in this study, we aimed to further investigate the inhibitory activity of FUBRS in its entirety on melanogenesis in B16F10 melanoma cells and to evaluate its molecular mechanism on the regulation of melanogenesis, including the p38, ERK and Akt signaling pathways.

## Materials and Methods

### Chemicals and Reagents

All media for cell growth, such as Dulbecco’s modified Eagle’s medium (DMEM) high glucose, fetal bovine serum (FBS) and trypsin-EDTA (0.25%) were purchased from Gibco-BRL Inc., USA, while 3-(4,5- dimethylthiazol-2-yl)-2,5-diphenyltetrazolium bromide (MTT) and 2,2-diphenyl-1-picrylhydrazyl (DPPH) were purchased from Sigma-Aldrich, USA. Trizol reagent was purchased from Invitrogen, USA. Kojic acid and L- DOPA were purchased from Tokyo Chemical Industry Co., Ltd., Japan. The anti-tyrosinase, anti-TYRP-2, anti- phospho-p38, anti-p38, anti-ERK1/2, and anti-phospho-ERK1/2 antibodies were purchased from Elabscience Biotechnology Co., Ltd., China.

### Isolation and Identiﬁcation of Microorganisms in the Loog-Pang E11 Microbial Starter

Appropriate serial dilutions of the loog-pang E11 microbial starter from Chiang Rai province, Thailand, were spread on potato dextrose agar plates for molds and yeast malt extract agar for yeasts and incubated at 30°C for 48-72 h. The LAB were isolated on De Man Rogosa and Sharpe agar with 0.04% (w/v) bromocresol purple and incubated at 37°C under a candle jar. The number of colonies was then counted and expressed as the number of colony-forming units per gram (CFU/g) of culture. The strains of molds, yeasts and LAB were identified by sequencing of the internal transcribed spacer (ITS) region using the ITS1 and ITS4 primers for molds and yeasts, and the 16S rRNA gene fragment using the 27F and 1492R primers for LAB [23]. Genomic DNA from each strain was isolated as previously described [22, 24] and used as the template for ITS or 16S rRNA amplification. DNA amplification was performed in a thermocycler with an initial denaturation step for 5 min at 95°C, followed by 35 cycles of denaturation for 30 sec at 95°C, annealing for 30 sec at 55°C, and extension for 1 min at 72°C and then a final extension for 5 min at 72°C. PCR products were sequenced by Macrogen, South Korea. The isolates were identified by matching each sequence to that giving the highest maximum identity score from the NCBI GenBank database using the Basic Local Alignment Search Tool (BLASTn). The sequences of the isolates showed 99.52%, 100%, 99.58%, and 97.58% sequence similarity to *Rhizopus oryzae* strain HP25, *Saccharomycopsis fibuligera* strain PPRI13335, *Saccharomyces cerevisiae* strain KSD-Yc, and *Pediococcus pentosaceus* strain IMAU50390, respectively. The sequences of the isolates were deposited in GenBank with accession numbers MN307112, MN307113, MN299328, and MN294560 for *R. oryzae* strain E1101, *Sm. fibuligera* strain E1102, *S. cerevisiae* strain E1103 and *P. pentosaceus* strain E1104, respectively.

### Sample Preparation

Fourty grams of raw UBR purchased from L H Rice International Co., Ltd. (Thailand) was mixed with distilled water at 1:2 (w/v) and autoclaved at 121°C for 15 min. The cooked UBR was cooled to room temperature and then fermented as follows: the cooked UBR was mixed with the loog-pang E11 starter culture at 2% (w/w) and incubated at 30°C in a closed sterilized bottle for 12 d. The obtained liquid (rice sap) from the rice fermentation (FUBRS) was collected and kept at -20°C for further study. The residual rice was extracted with hot water as previously described [25]. In brief, 1 g of rice residual was mixed with 10 ml of distilled water and boiled for 15 min. The sample was then clarified by centrifugation at 11,000 ×*g* for 15 min. The supernatant was kept at -20°C for further study.

### Cell Culture

B16F10 murine melanoma cell line (ATCC CRL-6475, Lot 63048505) and Hs68 human fibroblast cell line (ATCC CRL-1635, Lot 63691796) were purchased from the American Type Culture Collection (ATCC, USA). The cell lines were certified from ATCC to be mycoplasma contamination undetected. The cell lines were cultured in complete medium [CM; DMEM supplemented with 10% (v/v) FBS, penicillin (100 U/ml), and streptomycin (100 mg/ml)] at 37°C under a humidiﬁed 95% air, 5% CO_2_ atmosphere. The maintenance and culture of the cell lines were performed according to international guidelines on good cell culture practice [26].

### In Vitro Antioxidant Activity Assay

The antioxidant activity of the FUBRS was determined using the DPPH radical scavenging activity as previously described [27] with modification. The FUBRS at different concentrations and 10 μM ascorbic acid were separately incubated with 25 mg/l DPPH solution for 30 min and then the absorbance of the mixed solution at 515 nm (A_515_) was measured. The antioxidant activity was calculated as the DPPH scavenging activity as follows: DPPH scavenging activity (%) = [(A-B) ÷ A] × 100, where A and B are the A_515_ of H2O (as negative control) and the sample, respectively.

### Cellular Melanin Content Measurement

Cellular melanin content was measured as previously described (12). B16F10 cells were plated in 6-well plates (5× 104 cells/well) and incubated at 37°C under a humidiﬁed 95% air, 5% CO_2_ atmosphere for 24 h. The cells were then treated with FUBRS and incubated under same condition for 72 h. After incubation, cells were harvested and solubilized in 1 N sodium hydroxide at 60°C for 60 min, and the absorbance of the cell suspension was then measured by spectrophotometer at 405 nm (A_405_). The melanin content was expressed as the relative residual melanin (%) as follows: relative residual melanin (%) = [(A÷B) ÷ (C÷D)] × 100, where A and C are the A_405_ values of the treated and untreated cells, respectively, and B and D are the protein concentrations of the treated and the untreated cells, respectively. One mM of kojic acid (Tokyo Chemical Industry Co., Ltd., Japan) was used as a positive control.

### Cell Viability Assay

The relative cell viability was determined by the MTT assay. B16F10 cells (1 × 103 cells/ well) and Hs68 cells (1 × 105 cells/well) were plated in 96-well plates and incubated at 37°C under a humidiﬁed 95% air, 5% CO_2_ atmosphere for 24 h. The cells were then treated with varying concentrations of FUBRS [1, 2.5 and 5% (v/v)] and incubated at the same condition for 72 h. Then, 10 μl of 5 mg/ml MTT solution was added and incubated at 37°C for 4 h. After incubation, 100 μl of isopropanol was added and the absorbance at 540 nm (A_540_) was measured. The relative cell viability was then calculated as follows: cell viability (%) = (A÷B) × 100, where A is the A_540_ value of the treated cells and B is the A_540_ value of the untreated cells.

### Intracellular Tyrosinase Activity

Tyrosinase activity was determined as the L-DOPA oxidase activity as previously described [28]. B16F10 cells were plated in 6-well plates (5 × 10^4^ cells/well) and incubated at 37°C under a humidiﬁed 95% air, 5% CO_2_ atmosphere for 24 h. The cells were then treated with FUBRS and incubated under the same condition for 72 h. Then, the cells were washed with ice-cold phosphate-buffered saline (PBS) and completely lysed in lysis buffer [PBS containing 1% (v/v) Triton X-100]. After centrifugation at 18,000 ×*g* for 10 min, the supernatants were harvested and determined for tyrosinase activity. To this end, 90 μl of each lysate was mixed with 0.1% (w/v) L- DOPA (Tokyo Chemical Industry Co., Ltd.) and incubated at 37°C for 1 h. After that, the absorbance of the mixed solution was measured at 475 nm (A_475_) and determined as the relative tyrosinase activity (%) from the following equation: relative tyrosinase activity (%) = [(A÷B) ÷ (C÷D)] × 100, where A and C are the A_475_ values of the treated and untreated cells, respectively, and B and D are the protein concentrations of the treated and untreated cells, respectively.

### Two-Step Quantitative Real-Time Reverse Transcriptase (qrt-RT)-PCR

B16F10 cells were plated in 6-well plates (5 × 10^4^ cells/well) and incubated at 37°C under a humidiﬁed 95% air, 5% CO_2_ atmosphere for 24 h. The cells were then treated with 5% (v/v) of FUBRS and incubated under the same condition for 12 h. Total RNA was then extracted using Trizol solution (Invitrogen, USA) according to the manufacturer’s instruction. For the first-step RT-PCR, 1 μg total RNA was converted into complementary DNA (cDNA) using RevertAid Reverse Transcriptase (Thermo Fisher Scientific, USA). Then, the second-step qrt-PCR reaction was performed using the SsoAdvanced Universal SYBR Green Supermix (Bio-Rad, USA). The plates containing the reaction mixture were transferred to the CFX96 real-time system (Bio-Rad). Thermal cycling was performed at 95°C for 3 min, followed by 40 cycles of 95°C for 30 sec, primer specific temperature (49-55°C) for 30 sec (see Table 1) and 72°C for 30 sec. The specific primers used for determination of MITF, tyrosinase, TYRP1, TYRP2 and β-actin gene (as a loading control) transcript levels are shown in Table 1. The relative gene expression levels were calculated using the 2^−ΔΔCT^ method [29].

### Protein Quantification by Western Blot Analysis

B16F10 cells were plated in 6-well plates (5 × 10^4^ cells/ well) and incubated at 37°C under a humidiﬁed 95% air, 5% CO_2_ atmosphere for 24 h. The cells were then treated with FUBRS and incubated under the same condition for 72 h. After incubation, the total protein was extracted in RIPA solution [50 mM Tris-HCl pH 7.5, 150 mM NaCl, 1% (v/v) NP-40, 1% (w/v) Na-deoxycholate, 0.1% (w/v) sodium dodecyl sulfate (SDS)]. Total protein was separated by SDS-polyacrylamide gel electrophoresis (PAGE) with a 12% (w/v) acrylamide resolving gel and then transferred to a PVDF membrane. After blocking with blocking buffer [5% (w/v) skimmed milk in PBS with 0.1% (v/v) Tween 20 (PBST)], the membrane was incubated with primary antibody (anti-tyrosinase, anti-TYRP2, anti- p-ERK1/2, anti-ERK1/2, anti-p-P38, anti-P38, anti-p-Akt, anti-Akt, or anti-GAPDH) (Elabscience Biotechnology Co., Ltd., China) overnight at 1:1000 dilution in blocking buffer. After washing in PBST, the membrane was incubated in anti-rabbit IgG-horseradish peroxidase (HRP) (Cell Signaling Technology, USA) as the secondary antibody at a 1:2000 dilution in blocking buffer. After washing in PBST, immunoreactive bands were visualized with ECL solution (Thermo Fisher Scientific). The band densities were quantified using Bio-Rad Laboratories Quantity One 1-D Analysis Software (V 4.6; Bio-Rad). The relative band densities were determined using those of glyceraldehyde 3-phosphate dehydrogenase (GAPDH) as loading control. Data were expressed as a relative protein expression level.

### Statistical Analysis

Numerical data are shown as the mean ± one standard error of the mean (SEM) from the indicated number of independent repeats. The statistical significance of differences was analyzed using the GraphPad Prism by one- way analysis of variance (ANOVA), followed by Tukey's multiple comparison test (TMCT). A *p*-value < 0.05 was accepted as significant.

## Results

### UBR Fermented with Defined Starter E11 Decreased Melanin Content in B16F10 Melanoma Cells

**Fig. 1:**
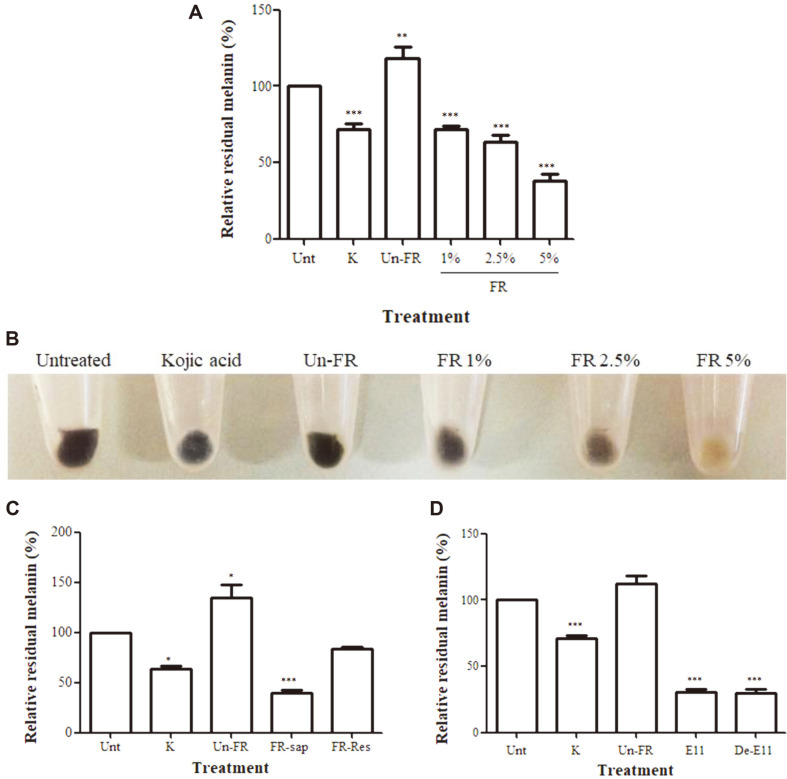
Reduction of melanin content in B16F10 melanoma cells by FUBRS. >B16F10 melanoma cells were treated with 1 mM kojic acid (K) as a positive control, unfermented UBR (Un-FR) at 5% (v/v) and FUBRS at 1, 2.5, and 5% (v/v) from fermentation with defined E11. (**A**) Melanin contents. (**B**) Visual observation of B16F10 melanoma cells after FUBRS treatment. (**C**) Residual melanin content in B16F10 melanoma cells after treatment with 5% (v/v) FUBRS (FR-sap) or FUBR residual rice (FR-Res). (**D**) Residual melanin content in B16F10 melanoma cells after treatment with 5% (v/v) FUBRS obtained from loog-pang E11 (E11) or Defined E11 (De-E11). Data are shown as the mean ± SEM from three independent experiments performed in triplicate. Statistically significant differences compared with untreated cells (Unt) are indicated by *p < 0.05, **p < 0.01, and ***p < 0.001.

A preliminary screening of various rice cultivars and various sources of microbial starters from loog-pang [22, 30] for the ability of the obtained fermented rice products to reduce the melanin content in B16F10 melanoma cells was performed. It revealed that FUBRS, the sap from UBR (Hom Nin rice) fermented with loog-pang microbial starter E11, gave the highest reduction of melanin content (Figs. 1A and 1B), and this activity was found in the sap but not in the residual rice (Fig. 1C).

The FUBRS at 1%, 2.5%, and 5% (v/v) dramatically reduced the melanin content (*p* < 0.001) in B16F10 melanoma cells in a dose-dependent manner compared with the untreated cells. In contrast, the unfermented UBR at 5% (v/v) significantly increased the melanin content compared to the untreated cells (Figs. 1A and 1B).

The microorganisms contained in the loog-pang E11 were isolated and identified. The identified microbes included the mold *R. oryzae* strain E1101 at 1 × 10^4^ CFU/g, the yeasts *S. cerevisiae* strain E1103 and *Sm. fibuligera* strain E1102 at 1 × 103 and 2 × 10^4^ CFU/g, respectively, and the LAB *P. pentosaceus* strain E1104 at 3 × 108 CFU/g. The mixture of these isolated microbes was designated “defined starter E11” and was used as the fermentation starter for all experiments to produce the FUBRS.

To confirm the role of these isolated microbes from the loog-pang E11 starter culture, the cooked UBR was fermented with the defined starter E11 (*R. oryzae*, *S. cerevisiae*, *Sm. fibuligera,* and *P. pentosaceus* at a 1 × 104, 1 × 103, 2 × 104, and 3 × 10^8^ CFU/g) for 12 d at 30°C and the harvested ferment sap was tested for its ability to reduce the melanin content in B16F10 melanoma cells. The FUBRS prepared by fermentation of UBR with the defined starter E11 showed a similar ability to reduce melanin in B16F10 melanoma cells as that from the ferment with loog-pang E11 (Fig. 1D), and so fermentation by these microbes (defined starter E11) played a role in producing the active compound(s) that reduce the melanin content in B16F10 melanoma cells.

### Antioxidant Activity of the FUBRS

**Fig. 2:**
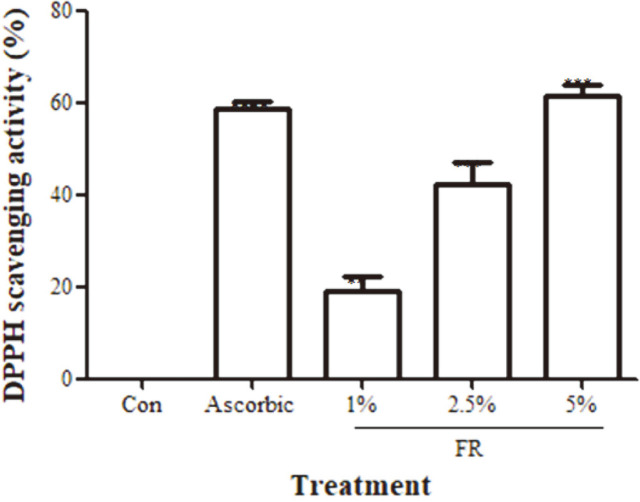
Dose-dependent antioxidant activity of FUBRS. The effect of varying concentrations of FUBRS (FR) and 10 μM of ascorbic acid (positive control) on the antioxidant activity were determined from the DPPH radical scavenging activity. Data are shown as the mean ± SEM from three independent experiments performed in triplicate. Statistically significant differences compared with H2O (Control; Con) are indicated by **p < 0.01 and ***p < 0.001.

Antioxidants protect cells from oxidative stress. The antioxidant capacity can be defined as the ability to scavenge ROS leading to a decrease in melanogenesis. To determine whether the FUBRS contained radical scavenging activities, we examined its ability to scavenge DPPH free radicals. For this, DPPH was incubated with the FUBRS at varying concentrations [0, 1, 2.5 and 5% (v/v)] and then the A_515_ was measured. The FUBRS showed a significant DPPH scavenging activity in a dose-dependent manner (Fig. 2), similar to that for ascorbic acid as the positive control (Fig. 2).

### Non-Cytotoxic Effect of FUBRS on the B16F10 Melanoma and Hs68 Human Fibroblast Cell Lines

**Fig. 3:**
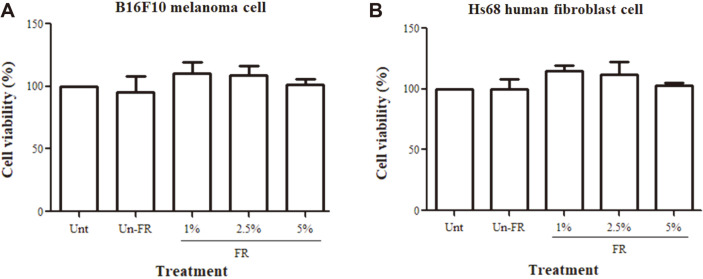
No cytotoxic effect of FUBRS against (A) B16F10 melanoma or (B) Hs68 human fibroblast cells. The cells were incubated with increasing concentrations of FUBRS (FR) or 5% (v/v) unfermented UBR (Un-FR). Relative cell viability (%) was determined by the MTT method compared to untreated cells (Unt). Data are shown as the mean ± SEM from three independent experiments performed in triplicate.

To determine the potential cytotoxicity of FUBRS on skin cells, B16F10 melanoma cells or Hs68 human fibroblast cells were treated with varying concentrations of FUBRS and then evaluated for their relative cell viability using the MTT assay. The results, expressed as the percent viability relative to the untreated control (Fig. 3), revealed that all tested concentrations of FUBRS and 5% (v/v) of unfermented UBR did not show any significant cytotoxicity to B16F10 melanoma (Fig. 3A) or Hs68 human fibroblast cells (Fig. 3B).

### Decreased Cellular Tyrosinase Activity by FUBRS

**Fig. 4:**
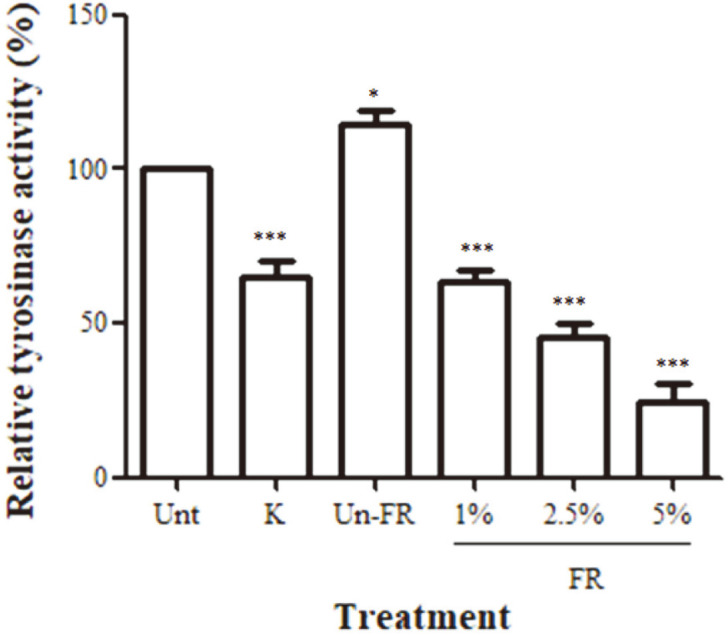
Decreased cellular tyrosinase activity induced by FUBRS. B16F10 melanoma cells were treated with 1 mM kojic acid (K) as a positive control, unfermented UBR (Un-FR) at 5% (v/v) and varying concentrations [1, 2.5, and 5% (v/v)] of FUBRS (FR) prior to determining the tyrosinase activity. Data are shown as the mean ± SEM from three independent experiments performed in triplicate. Statistically significant differences compared with untreated cells (Unt) are indicated by *p < 0.05 and ***p < 0.001.

Tyrosinase is the essential enzyme in melanogenesis. Thus, we examined the effect of FUBRS on cellular tyrosinase activity. B16F10 melanoma cells were treated with varying concentrations [1, 2.5, and 5% (v/v)] of FUBRS and then the cellular tyrosinase activity was measured. Kojic acid (1 mM) was used as a positive control. The relative tyrosinase activity of B16F10 melanoma cells treated with FUBRS at 1%, 2.5%, and 5% (v/v) was 63%, 45%, and 24%, respectively, relative to the untreated control (Fig. 4). A similar result was obtained with kojic acid-treated B16F10 cells (*p* < 0.001). In contrast, the unfermented UBR significantly stimulated cellular tyrosinase activity (*p* < 0.05) compared to the untreated cells (Fig. 4). Therefore, FUBRS significantly inhibited cellular tyrosinase activity in a dose-dependent manner (*p* < 0.001).

### Decreased Expression Level of Melanogenesis-Related Proteins by FUBRS

**Fig. 5:**
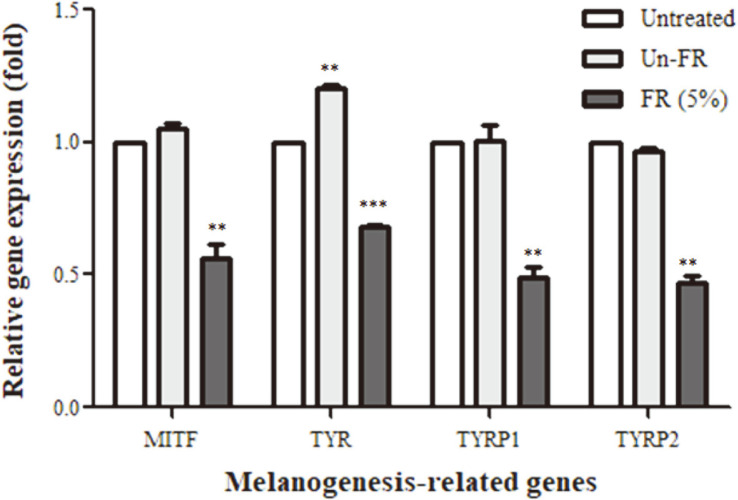
Reduction of melanogenesis-related gene expression levels by FUBRS. B16F10 melanoma cells were treated with 5% (v/v) unfermented UBR (Un-FR) or FUBRS (FR) and then the mRNA levels of MITF, tyrosinase, TYRP1, and TYRP2 were determined by two-step qrtRT-PCR. Values are the means ± SEM from three independent experiments performed in triplicate. Statistically significant differences compared with untreated cells (Unt) are indicated by **p < 0.01 and ***p < 0.001.

The expression of the melanogenesis-related proteins MITF, tyrosinase, TYRP1, and TYRP2 is essential for melanogenesis. Thus, to elucidate whether the FUBRS affected the expression levels of these melanogenesis-related proteins, we examined their transcript and protein expression levels in B16F10 melanoma cells after FUBRS treatment by two-step qrtRT-PCR and western blot analysis, respectively. Treatment with 5% (v/v) FUBRS suppressed the mRNA expression level of MITF and its downstream genes encoding for tyrosinase, TYRP1, and TYRP2 (Fig. 5). On the other hand, the unfermented UBR treatment significantly increased the mRNA expression level of tyrosinase and had no significant effect on the mRNA expression level of MITF, TYRP1, and TYRP2 compared to the untreated control (Fig. 5).

**Fig. 6:**
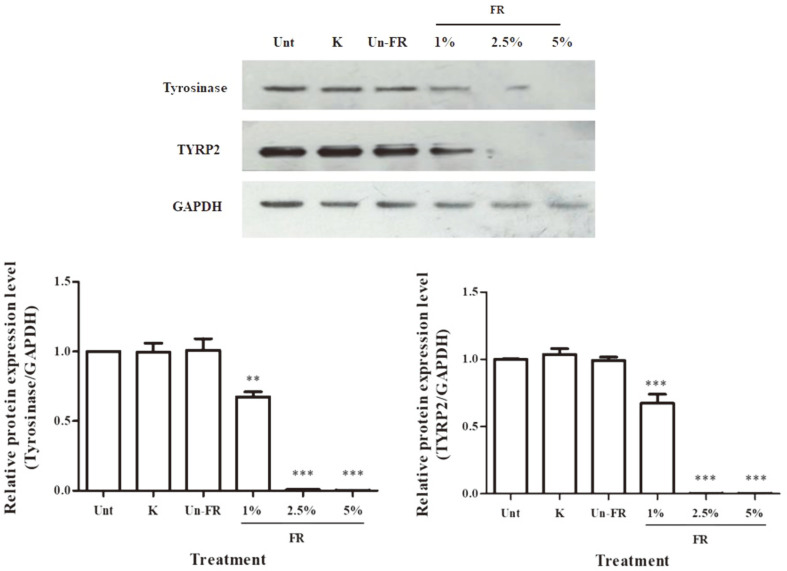
Effect of the FUBRS on the protein expression level of tyrosinase and TYRP2 in B16F10 melanoma cells. B16F10 melanoma cells were treated with 1 mM kojic acid (K) as a positive control, unfermented UBR (Un-FR) at 5% (v/v) and varying concentrations [1, 2.5, and 5% (v/v)] of FUBRS (FR) for 72 h. The protein expression levels were determined by western blot analysis and normalized to that of GAPDH. Values are the mean ± SEM from three independent experiments performed in triplicate. Statistically significant differences compared with untreated control (Unt) are indicated by symbols *p < 0.05, **p < 0.01, and ***p < 0.001.

In addition, the varying concentrations [1, 2.5, and 5% (v/v)] of FUBRS significantly reduced the protein expression levels of tyrosinase and TYRP2 (Fig. 6) in a dose-dependent manner (*p* < 0.001) compared to the untreated cells. It was noted, however, that the potent tyrosinase inhibitor, kojic acid, and the unfermented UBR did not affect the expression levels of tyrosinase and TYRP-2 (Fig. 6).

### Induced Phosphorylation of Erk1/2, p38 and Akt Signaling Pathways by FUBRS

**Fig. 7:**
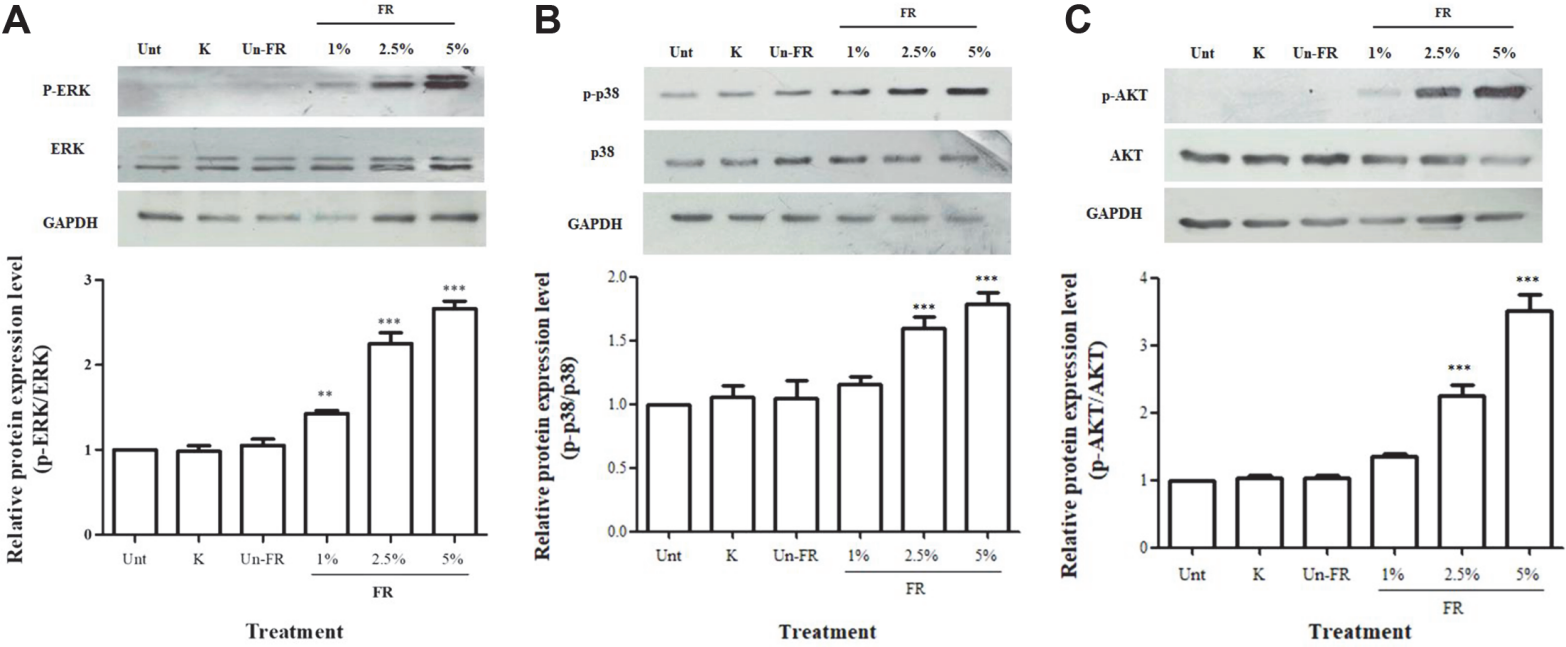
Effect of FUBRS on phosphorylation of ERK1/2 (p-ERK), p38 (p-p38), and Akt (p-Akt) in B16F10 melanoma cells. B16F10 melanoma cells were treated with 1 mM kojic acid (K), unfermented UBR (Un-FR) at 5% (v/v) and varying concentrations [1, 2.5, and 5% (v/v)] of FUBRS (FR) for 72 h. The phosphorylation levels of (**A**) ERK1/2, (**B**) p38, and (**C**) Akt were determined by western blot analysis and normalized to the expression of the respective non-phosphorylated protein. Values are the means ± SEM from three independent experiments performed in triplicate. Statistically significant differences compared with untreated control (Unt) are indicated by symbols **p < 0.01 and ***p < 0.001.

Several signaling pathways, including the MAPK (p38 and ERKs) and PI3K/Akt pathway, modulate melanin formation and melanogenic gene expression. To determine the potential modulation of these pathways by FUBRS, B16F10 melanoma cells were exposed to FUBRS and the phosphorylation of these proteins was determined by western blot analysis. The FUBRS-treated cells showed a significantly increased level of phosphorylated ERK1/2 (Fig. 7A), p38 (Fig. 7B), and Akt (Fig. 7C) in a dose-dependent manner (*p* < 0.001), whereas kojic acid and the unfermented UBR did not affect the level of phosphorylation of these proteins (Fig. 7).

## Discussion

Melanin plays a crucial role in protecting skin against the harmful effects from UV light. However, overproduction and accumulation of melanin can create skin problems, such as freckles, age pigment and melasma. Therefore, melanogenesis inhibition has been focused on as an effective method for skin depigmentation and lightening in medicinal and cosmeceutical applications [31, 32]. Currently, many compounds, such as arbutin, hydroquinone and kojic acid, are known as melanogenesis inhibitors and are used in skin whitening agents. However, they can induce skin disorders and possess mutagenesis properties [33-35]. Based on these side effects, the search for safer and more effective whitening agents, including from natural sources, is still ongoing in the field of cosmeceuticals research and development.

A previous study showed that fermented rice could reduce the activity of mushroom tyrosinase [36], an in vitro assay that is routinely used for screening for potential inhibitors of melanogenesis. However, many melanogenesis inhibitors obtained from screening against mushroom tyrosinase did not exhibit inhibitory effects on cellular tyrosinase activity (3). In addition, other important properties of a potential inhibitor that are not screened in this in vitro assay include the permeability of the compound into the cells, stability of the compound inside the cells and, especially, cytotoxicity of the compound [37]. Thus, in this study we aimed to elucidate the molecular mechanism of the FUBRS in its entirety on the anti-melanogenesis activity in the B16F10 melanoma cells.

We successfully produced FUBRS with a potent anti-melanogenesis activity (Fig. 1) using UBR (Hom Nin rice) as the substrate and fermenting it using the defined starter E11, containing *R. oryzae, S. cerevisiae*, *Sm. fibuligera*, and *P. pentosaceus*. These were originally isolated from the selected loog-pang E11, a starter of Thai rice-based fermented rice dessert, khao-mak [38] or Thai rice-based alcoholic beverage, Sato [22, 30]. In contrast, the unfermented UBR stimulated melanin production in the B16F10 melanoma cells (Fig. 1A), which is in agreement with a previous report that black rice (Hom Nin rice) stimulated melanogenesis [20].

In contrast to unfermented UBR, the FUBRS strongly inhibited melanogenesis in the B16F10 cells. Thus, during fermentation of UBR bioactive compound(s) could be produced leading to enhancement of various biological activities responsible for the inhibition of melanogenesis. Previously, it was reported that rice bran fermented with fungi or lactic acid bacteria could produce active ingredients or increase levels of active ingredients such as α-tocopherol, ascorbic acid, lactic acid, vanillic acid, caffeic acid, protocatechuic acid and 4- hydroxybenzoic etc. [25, 39], and may have an effect on melanogenesis inhibition. The positive effect of fermentation on increasing some bioactive compounds has been reported previously [40, 41].

Antioxidants that protect cells from oxidative stress are known to play pivotal roles in the inhibition of melanogenesis in B16F10 melanoma cells [42]. The FUBRS contained a dose-dependent antioxidant activity, as determined by the DPPH assay (Fig. 2). This may be from the anthocyanin contained in the UBR [43].

With respect to toxicity to the skin cells, FUBRS at the concentrations up to 5% (v/v), the highest concentration tested, showed no cytotoxic effect to B16F10 melanoma cells (Fig. 3A) or Hs68 human fibroblast cells (Fig. 3B), suggesting that it is safe for skin cells, and at least for melanoma cells and the fibroblast cells, and so potentially could be used in cosmeceuticals as a whitening agent.

As to the mechanism of inhibition of melanogenesis, FUBRS potently decreased cellular tyrosinase activity in a dose-dependent manner (Fig. 4), consistent with the dramatically decreased cellular melanin content in B16F10 melanoma cells (Fig. 1). Likewise, the fermented rice sap from purple plain rice was previously reported to inhibit mushroom tyrosinase activity (30). In this study, the FUBRS showed an inhibitory effect on tyrosinase activity (Fig. 4) and on the expression level of tyrosinase (Figs. 5 and 6). In contrast, the unfermented UBR showed the opposite effect, with a significant stimulation of tyrosinase activity (Fig. 4) and tyrosinase gene expression (Fig. 5). This contrasting effect between the fermented and unfermented UBR emphasized the important role of the defined starter E11 on transformation of the compound(s) in the rice substrate to form the biological activity.

The FUBRS significantly reduced the transcript expression level of MITF, a major transcription factor in the regulation of melanogenic enzyme that plays a critical role in melanin biosynthesis [44]. Likewise, FUBRS reduced the transcript level of enzymes downstream of MITF, including tyrosinase, TYRP1, and TYRP2 (Fig. 5), and significantly reduced the expression of tyrosinase and TYRP2 in a dose-dependent manner at the translational level (Fig. 6). In contrast, kojic acid, a well-known inhibitor of tyrosinase, did not affect the expression levels of tyrosinase, TRP-1, TRP-2, and MITF. These results suggested that FUBRS has different melanogenesis inhibition mechanisms from kojic acid. The FUBRS likely reduced melanogenesis by inhibiting tyrosinase activity and the expression levels of tyrosinase, TYRP1, and TYRP2 via inhibiting expression of MITF in B16F10 melanoma cells.

Previous studies have also shown that the MAPK pathway, including ERK and p38, play important regulatory roles during melanogenesis [45], where melanogenesis inhibitors activated the phosphorylation of ERK and p38. These phosphorylations resulted in the phosphorylation of MITF at serine 73 and subsequent ubiquitin- dependent proteasomal degradation [46]. Accordingly, we examined the phosphorylation level of ERK and p38 in B16F10 melanoma cell after treatment with FUBRS. The results revealed that FUBRS activated the phosphorylation of ERK and p38 (Figs. 7A and B).

Besides the MAPK pathway, PI3K/Akt causes MITF transcription to bind to the target sequence and induce melanogenesis. Hence, inhibition of the phosphorylation of Akt leads to the increasing of MITF expression resulting in melanin synthesis [47]. It has been reported that sesamol elevated the phosphorylation of Akt, possibly by reducing MITF transcription, and this led to the inhibition of melanin production and tyrosinase activity [48]. In our study, FUBRS significantly increased the level of phosphorylated Akt (Fig. 7C). Taken together, the results from Fig. 7A, 7B and 7C demonstrate that, FUBRS may reduce the level of MITF by increasing MITF degradation as a result from increased phosphorylation of ERK and p38 and also reduce MITF transcription as a result of increased phosphorylation of Akt.

**Fig. 8:**
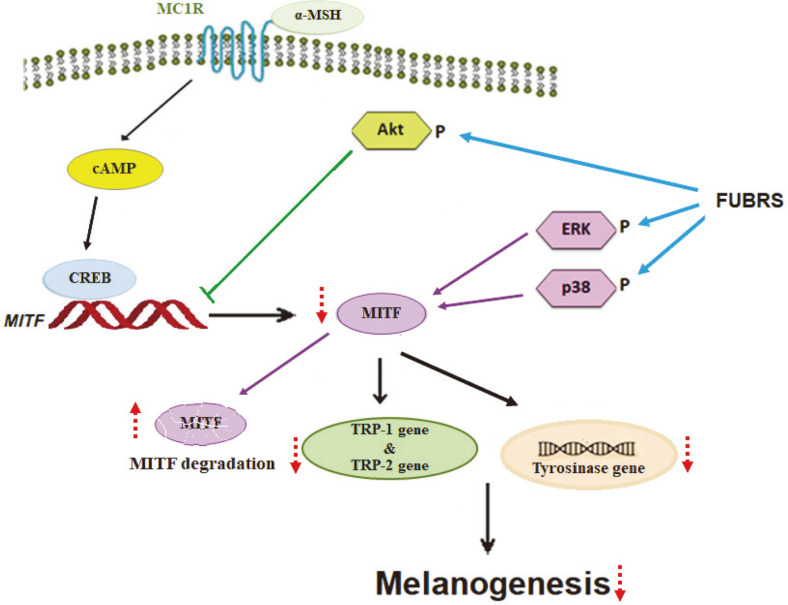
Schematic description of the inhibition of melanogenesis in B16F10 melanoma cells upon FUBRS treatment. Symbols: represents activation, represents inhibition, blue arrow represents the effect of FUBRS on the phosphorylation of ERK, p38 and Akt, red dash arrow represents results from the phosphorylation of ERK, p38 and Akt. The FUBRS stimulated phosphorylation of ERK and p38 leading to MITF degradation (purple line) and it also stimulated phosphorylation of Akt (green line) leading to the decreasing of MITF expression. The results from increasing phosphorylation of ERK, p38 and AKT led to decrease in the MITF level, thus, the expression of genes downstream of MITF such as tyrosinase, TYRP1, TYRP2 were decreased.

The present study showed that the fermentation of UBR (Hom Nin rice) with the defined mixed culture of *R. oryzae, S. cerevisiae*, *Sm. fibuligera*, and *P. pentosaceus* to form FUBRS resulted in a potent reduction of cellular melanin content and antioxidant activity. The FUBRS had an antioxidant activity that may have the ability to scavenge ROS. It is known that ROS stimulates alpha-melanocyte stimulating hormone production in keratinocytes and binds to the melanocortin 1 receptor on melanoma cells leading to melanogenesis [49, 50]. In addition, it inhibited melanin biosynthesis by stimulating phosphorylation of ERK and p38 leading to MITF degradation and also stimulating phosphorylation of Akt leading to a reduction of MITF expression. As a consequence, the expression of genes downstream of MITF including TYRP1, TYRP2, and tyrosinase genes, was reduced. A schematic diagram of the potential mechanisms of FUBRS on melanogenesis is summarized in Fig. 8.

The mechanism for the melanogenesis inhibition by FUBRS appears to be more complex than that of kojic acid, the known tyrosinase inhibitor. Therefore, the isolation and identification of major bioactive compounds from the FUBRS crude extract would be the next step of our study. This is under investigation and will be published elsewhere.

Overall, the FUBRS obtained in this study has high potential for further cosmeceuticals application as a depigmenting agent. The role of the defined starter E11 on producing bioactive compounds with anti-melanogenesis is also under investigation.

## Figures and Tables

**Table 1:** Oligonucleotide primers used for two-step quantitative real-time reverse transcriptase (qrt-RT)-PCR.

Primer name	Primer sequence (5'–3')	Annealing temperature	Product size (bp)
MITF	GTATGAACACGCACTCTCGA (Forward)	49°C	135
	GTAACGTATTTGCCATTTGC (Reverse)		
Tyrosinase	GTCGTCACCCTGAAAATCCTAACT (Forward)	52°C	111
	CATCGCATAAAACCTGATGGC (Reverse)		
TYRP1	CTTTCTCCCTTCCTTACTGG (Forward)	52°C	163
	TCGTACTCTTCCAAGGATTC (Reverse)		
TYRP2	TTATATCCTTCGAAACCAGGA (Forward)	52°C	176
	TTATATCCTTCGAAACCAGGA (Reverse)		
β-actin	ATGGAGT (Forward)	55°C	105
	CAGGGCAGTGATCTCCTTC (Reverse)		
